# COVID‐19 and the liver – Lessons learned

**DOI:** 10.1111/liv.14854

**Published:** 2021-06-21

**Authors:** Toni Herta, Thomas Berg

**Affiliations:** ^1^ Division of Hepatology Department of Medicine II Leipzig University Medical Center Leipzig Germany; ^2^ Department of Gastroenterology and Hepatology Tytgat Institute for Liver and Intestinal Research AGEM Amsterdam University Medical Centers Amsterdam the Netherlands

## Abstract

Liver involvement, indicated by elevated liver function test results, is common in hospitalized patients with coronavirus disease 2019 (COVID‐19) and has been linked to disease severity and outcome. A dual pattern of elevated liver function tests can be observed especially in patients with severe or critical COVID‐19, characterized by an increase in aminotransferases early in the course of this disease, followed by an increase in cholestasis‐associated biochemistry markers at later stages. This dual pattern is associated with inflammatory response markers and poor outcome. Current notions on the mechanisms of liver injury in COVID‐19 include direct cytopathic effects of the virus on hepatocytes and cholangiocytes, ischemic and hypoxic liver damage, drug‐induced liver injury, activation of hepatic immune cells by excess cytokine production and exacerbation of pre‐existing liver disease. Patients with obesity‐related non‐alcoholic fatty liver disease and, in particular, patients with cirrhosis are at high risk of liver injury and a fatal outcome from COVID‐19. In contrast, individuals receiving stable immunosuppressive medication for autoimmune liver diseases or during long‐term follow‐up after liver transplantation do not have a higher case‐to‐infection ratio and have a fairly favourable outcome. The present review describes the epidemiology, characteristics and potential pathological mechanisms of COVID‐19‐related liver injury. Moreover, the influence of pre‐existing liver disease on the susceptibility and severity of liver injury in COVID‐19 are discussed.

**Abbreviations**
ACE2: angiotensin‐converting enzyme 2
ACLF: acute‐on‐chronic liver failure
AIH: autoimmune hepatitis
ALAT: alanine aminotransferase
AP: alkaline phosphatase
ARDS: acute respiratory distress syndrome
ASAT: aspartate aminotransferase
COVID‐19: coronavirus disease 2019
CRP: C‐reactive protein
CTP: Child‐Turcotte‐Pugh
GGT: gamma‐glutamyl transferase
HBV: hepatitis B virus
HCV: hepatitis C virus.
IL: interleukin
INR: international normalized ratio
LT: liver transplantation
MELD: model for end‐stage liver disease
NAFLD: non‐alcoholic fatty liver disease
NAS: NAFLD activity score
NASH: non‐alcoholic steatohepatitis
NSAID: non‐steroidal anti‐inflammatory drug
PBC: primary biliary cholangitis
PSC: primary sclerosing cholangitis
RNA: ribonucleic acid
SARS‐CoV‐2: severe acute respiratory syndrome coronavirus 2
TMPRSS2: transmembrane protease serine 2
ULN: upper limit of normal

**Key points**

- Liver involvement, characterized by elevated liver function tests, is common in hospitalized patients with COVID‐19.
- Patients with severe or critical disease courses are more likely to have elevated liver function tests and higher peaks of elevation.
- The mechanism of elevation is probably multifactorial liver injury.
- Patients with obesity‐related non‐alcoholic fatty liver disease and, in particular, patients with advanced cirrhosis are at high risk of liver injury and a fatal outcome from COVID‐19.
- Optimal treatment and compensation of chronic liver disease are critical to prevent severe courses of COVID‐19 in these patients.

## 1 INTRODUCTION

Coronavirus disease 2019 (COVID‐19) is a contagious, zoonotic respiratory infection caused by severe acute respiratory syndrome coronavirus 2 (SARS‐CoV‐2). COVID‐19 was first reported in December 2019 in a series of patients with severe pneumonia with a fatal outcome in certain cases, following exposure to the Huanan seafood market in Wuhan, Central China,^1^ and has since spread worldwide with more than 37 million cases and 1 million deaths, as of October 2020. In addition to acute respiratory tract symptoms, abnormal liver function tests were observed in 14%‐69% of patients, mostly identified by transient elevation of aminotransferases.^2^, ^3^, ^4^ Liver injury from COVID‐19 seems to mirror disease severity, as patients with severe COVID‐19 are more likely to have elevated liver function tests^3^, ^5^ and higher peaks of elevation^4^, ^6^ than those with milder disease. Whether these observations reflect direct SARS‐CoV‐2‐mediated liver damage, secondary liver injury from systemic COVID‐19 or more severe courses of COVID‐19 in patients with pre‐existing liver disease has not been clarified. The present review describes the epidemiology, characteristics and potential pathological mechanisms of COVID‐19‐related liver injury. Moreover, the influence of pre‐existing liver disease on the susceptibility to, severity of and liver injury in COVID‐19 are discussed.

## 2 ABNORMAL LIVER FUNCTION TESTS AS RISK FACTOR FOR SEVERE COVID‐19

**FIGURE 1:**
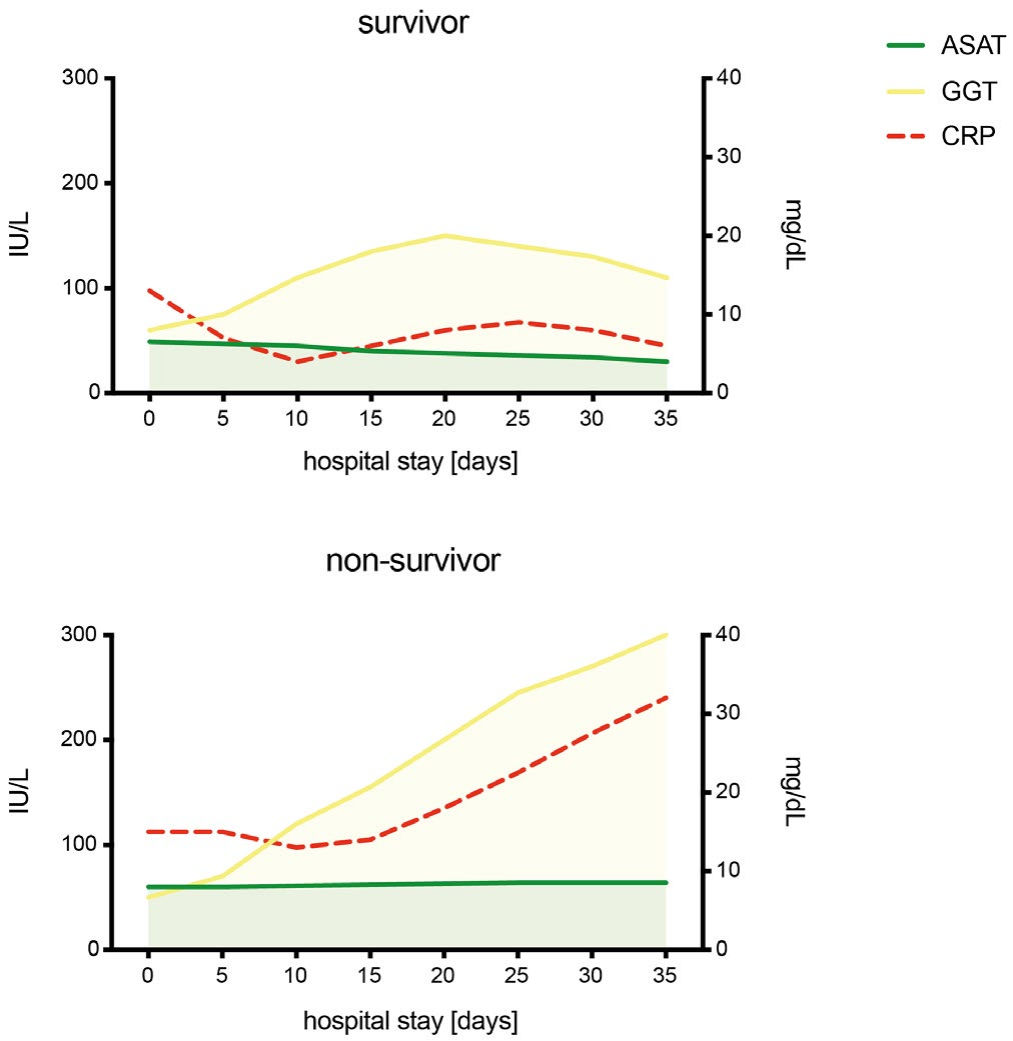
Trends of ASAT, GGT and CRP in survivors and non‐survivors of COVID‐19. Longitudinal variations were extrapolated from a median of 3 (1‐15) laboratory tests in 540 patients (survivors n = 431, non‐survivors n = 109). ASAT, aspartate aminotransferase (IU/L, left coordinate); GGT, gamma‐glutamyl transferase (IU/L, left coordinate); CRP, C‐reactive protein (mg/dL, right coordinate). Adapted from Ref. [9] [Correction added on 5 August 2021, after first online publication: Figure 1 image has been replaced.]

Abnormal liver function tests in patients with COVID‐19 were first reported in a cohort of 99 patients at Jinyiantan Hospital in Wuhan. Nearly all patients (98%) presented with decreased albumin levels (mean 31.6 g/L, normal range 40‐55 g/L). Alanine aminotransferase (ALAT) and aspartate aminotransferase (ASAT) levels were moderately elevated in 28% and 35% of patients respectively. One patient had severe liver damage (ALAT 7590 U/L and ASAT 1445 U/L). Slightly elevated total bilirubin levels were less common (18% of the cases).^7^ Twelve single‐ and multicentre studies in China with a total of 2264 included patients analysed aminotransferase levels in patients with COVID 19.^2^ Aminotransferase levels were above the upper limit of normal (ULN) at least once in 14%‐53% of the patients. Interestingly, the proportion of patients with increased aminotransferase levels was higher in Wuhan, the epicentre of COVID‐19, than outside Wuhan (21% vs 10%, *P* < .0001).^3^, ^8^ The authors suggest that this could be because of exposure to higher doses of SARS‐CoV‐2 with more severe courses of COVID‐19 in Wuhan. A meta‐analysis of 35 studies including 6686 patients evaluated elevated liver function tests in relation to the severity of COVID‐19. ALAT, ASAT and total bilirubin levels were significantly higher in patients with severe COVID‐19 than in those with non‐severe disease (odds ratio 1.89 [*P* = .0009], 3.08 [*P* < .0001] and 1.39 [*P* < .0001] respectively).^3^ In a study in 417 patients, abnormal liver tests on hospital admission were classified as hepatocellular (ALAT and/or ASAT > 3× ULN), cholestatic (alkaline phosphatase (AP) and/or gamma‐glutamyl transferase (GGT) > 2× ULN) or mixed. Patients with hepatocellular‐ and mixed‐ but not cholestatic‐type abnormal liver function tests upon admission had a significantly higher risk of developing severe pneumonia than those without any abnormalities (odds ratio 2.73 [*P* = .02] and 4.44 [*P* < .001]).^6^ The authors concluded that liver test abnormalities upon hospital admission, in particular, elevated ALAT or ASAT, can be used to predict the severity of COVID‐19. Elevation was usually (>90% of the cases) mild on admission (<2× ULN), and increased in 24% of the cases to significantly more than 3× ULN during hospitalization, again associated with the severity of COVID‐19 pneumonia (odds ratio 3.19 [95% confidence interval 1.15‐8.84] for hepatocellular and 11.22 [95% confidence interval 4.42‐28.45] for mixed type).^6^ A study at Massachusetts General Hospital in the USA followed liver function tests in 60 patients with COVID‐19 for a median of 9 days during hospitalization. Aminotransferases increased to >ULN in 93% of the patients, while AP and total bilirubin levels remained normal (AP) or were mildly elevated (total bilirubin) in most patients, consistent with hepatocellular injury. Aminotransferases were >5× ULN in 17% of the patients. In particular, ASAT was higher than ALAT at admission (46 vs 30 U/L) and for most of the hospital stay (*P* < .05). Peak ASAT levels were higher in patients requiring mechanical ventilation (*P* = .003) and correlated with the length of hospital stay (*P* = .03).^4^ An interesting, dual pattern of liver damage was reported in a survey including 540 hospitalized patients with severe COVID‐19 from Zaragoza, Northern Spain, in which 40.9% and 47.3% of the patients presented with elevated ASAT and GGT levels at admission respectively. There was a negative correlation between initial oxygen saturation and ASAT but not GGT (*P* <.001 and *P* =.944 respectively). A longitudinal analysis showed that the progression of GGT levels was positively correlated with inflammatory markers such as C‐reactive protein (CRP) and strongly increased in non‐survivors but not in survivors during hospitalization (*P* < .001).^9^ The authors concluded that SARS‐CoV‐2 may have a dual effect on the liver, characterized by elevated aminotransferases on admission followed by a marked cholestasis in patients with a fatal outcome (Figure 1). Median albumin levels were already lower on admission in non‐survivors than in survivors (3 g/dL vs 3.4 g/dL, respectively, *P* < .001), and further decreased in these patients during hospitalization.^9^ In conclusion, abnormal liver function tests are common in COVID‐19, mainly in the form of transient increases in aminotransferases. The incidence is higher in patients with severe COVID‐19 than in those with mild disease. Acute hepatitis is occasionally reported. In patients with severe COVID‐19, initial liver injury is characterized by elevated aminotransferases followed by a cholestatic pattern, and a significant decrease in albumin later in the course of the disease. Studies of liver function tests in outpatients with COVID‐19 are lacking.

## 3 PATHOGENESIS AND CAUSES OF HEPATIC COMPLICATIONS DURING SARS‐COV‐2 INFECTION

**FIGURE 2:**
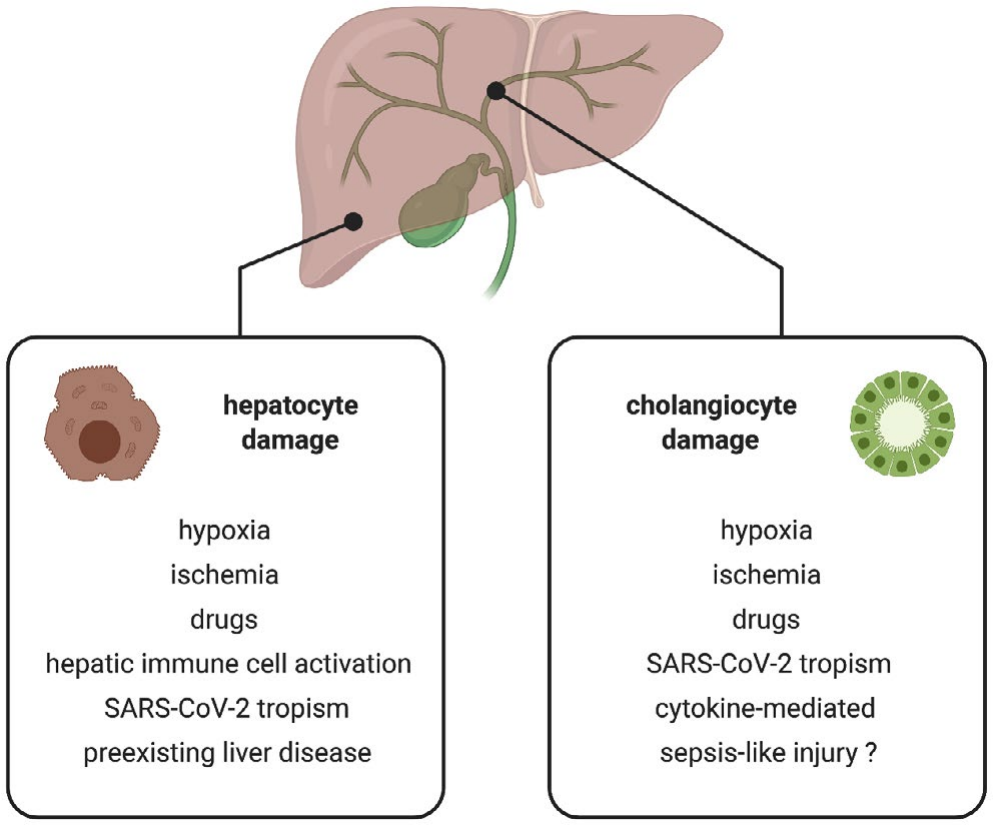
Potential mechanisms of hepatocyte and cholangiocyte injury in COVID‐19. SARS‐CoV‐2, severe acute respiratory syndrome coronavirus 2 [Correction added on 5 August 2021, after first online publication: Figure 2 image has been replaced.]

Reports on the mechanism of COVID‐19‐related liver injury are limited. However, several notions have been discussed (Figure 2): (i) *Direct SARS‐CoV‐2‐induced cytopathic effects on hepatocytes and cholangiocytes*. SARS‐CoV‐2 expresses a surface glycoprotein, called spike, on the viral envelope, which binds to the receptor angiotensin‐converting enzyme 2 (ACE2) on human host cells and, thus, mediates viral entry into the host cell cytoplasm.^10^ ACE2 is expressed in a variety of human tissues such as the lungs, heart, kidneys, pancreas, blood vessels, adipose tissue and liver. Single‐cell RNA sequencing revealed marked enrichment of ACE2 expression in cholangiocytes but an average expression in hepatocytes that was 20 times lower (59.7% vs 2.6% of ACE2 positive cells).^11^ Hepatic immune and stromal cells were ACE2 negative. Thus, it could be hypothesized that SARS‐CoV‐2 infects cholangiocytes but probably not hepatocytes. However, this notion is not supported by the cell tropism profile of SARS‐CoV‐2. Human Huh7 hepatocellular carcinoma cells are highly susceptible to SARS‐CoV‐2 infection.^12^ Moreover, liver and cholangiocyte organoids derived from human pluripotent stem cells are permissive to SARS‐CoV‐2 infection as seen by high levels of viral RNA transcription after inoculation with a SARS‐CoV‐2 isolate. Infected hepatocytes and cholangiocytes showed marked pro‐inflammatory chemokine induction and downregulation of metabolic markers.^13^ Viral genomic RNA was identified in 3 of 4 samples of a series of post‐mortem examinations of the livers of patients who died from severe COVID‐19.^14^ One possible explanation for the unexpected tropism of SARS‐CoV‐2 to hepatocytes could be its exceptionally strong binding affinity to ACE2 which could facilitate virus entry despite low ACE2 expression levels.^10^, ^12^ Furthermore, as discussed below, hypoxia and pre‐existing liver disease are thought to induce hepatocellular ACE2 expression and potentially increase hepatic susceptibility to SARS‐CoV‐2 infection.^15^, ^16^ One study observed spike structures in the cytoplasm of hepatocytes of 2 COVID‐19 cases with transmission electron microscopy. These structures were defined as coronavirus particles. Affected hepatocytes exhibited potentially cytopathic lesions such as mitochondrial swelling, endoplasmatic reticulum dilatation and a decrease in glycogen granules. Furthermore, hepatocellular apoptosis and syncytialization were observed.^17^ However, in contrast to the histopathological findings, these two cases did not fulfil the clinical criteria of acute liver failure. Furthermore, the observed changes may be seen during multi‐organ dysfunction associated with critical illness, drug‐induced liver injury and fatty liver disease, as described in.^18^ It was also suggested that the spiked, ‘corona‐like’ inclusions may have been intrahepatic cholesterol crystals or ‘crown‐like’ structures seen in patients with fatty liver disease.^18^ In conclusion, SARS‐CoV‐2 may target cholangiocytes and hepatocytes through ACE2 but the extent of cytopathic damage and liver injury caused by this potential infection remains to be clarified. (ii) *Complex immune dysregulation and hypoxic liver injury*. Patients with severe COVID‐19 display a unique signature of immune dysregulation with two key features: overproduction of pro‐inflammatory cytokines by monocytes and dysregulation of lymphocytes with lymphopenia.^19^, ^20^, ^21^ SARS‐CoV‐2 may trigger a hyperinflammatory syndrome, called macrophage activation syndrome or secondary haemophagocytic lymphohistocytosis in a subset of patients with severe COVID‐19.^20^, ^21^ This syndrome is characterized by excessive release of cytokines (“cytokine storm”), cytopenias, disseminated intravascular coagulation and multiple organ dysfunction (including the lungs and liver). Interleukin (IL)‐6 signalling plays a central role in the pathophysiology of cytokine‐driven hyperinflammatory syndromes.^20^ In a study from Austria, serum IL‐6 levels were strongly correlated with elevated ASAT levels and peak ASAT and ALAT elevation in 96 hospitalized patients with COVID‐19.^22^ This correlation was stronger in patients with severe COVID‐19 than in those with non‐severe disease (coefficient of determination *r* ^2^ .610 vs .481, *P* < .05). Circulating cytokines can induce a transient elevation of aminotransferases (eg by activation of hepatic immune cells) without affecting liver function, a phenomenon called “bystander hepatitis”, which is often observed in systemic viral infections.^23^ Moreover, hyperinflammatory syndromes can induce disseminated intravascular coagulation with ischemic and hypoxic liver damage by microvascular thrombosis. Hypoxia and ischemia are probably potentiated by respiratory insufficiency with hypoxemia and haemodynamic alterations.^8^, ^24^ High levels of positive end‐expiratory pressure in patients with COVID‐19 who require mechanical ventilation probably further impairs hepatic perfusion by impeding venous drainage.^25^ Post‐mortem liver biopsies of patients with fatal COVID‐19 showed microvesicular steatosis, hepatocellular degeneration, lobular focal necrosis, portal immune cell infiltration and microthrombosis with congestion of the hepatic sinuses—findings that are consistent with ischemic or hypoxic liver damage.^6^, ^24^ Preliminary autopsy results from Bergamo, Italy, suggest that partial or complete sinusoidal or portal thrombosis are common in cases of fatal COVID‐19 with elevated aminotransferases, and were found in 27% and 73% of the analysed samples respectively.^26^ Changes in coagulation‐related biomarkers, such as elevated D‐dimer levels, are consistently found in patients with COVID‐19, and are more pronounced in critically ill cases.^27^ Besides disseminated intravascular coagulation, SARS‐CoV‐2 may promote endothelial cell injury in the arteries, veins, arterioles, capillaries and venules of all major organs, which probably further impairs hepatic microcirculation and promotes thrombus formation.^28^, ^29^ In ischemic or drug‐induced liver injury, ASAT levels usually peak before ALAT levels, a pattern that is often observed in patients with severe COVID‐19.^4^, ^22^ In conclusion, abnormal liver function tests in COVID‐19 may be a result of a severe inflammatory immune response, either as a result of “bystander hepatitis” or ischemic (hypoxic) liver damage from microvascular thrombosis, hypoxemia and altered hepatic perfusion. (iii) *Drug‐induced liver injury*. The list of drugs with potentially hepatotoxic effects that are used or have been tested for the treatment of patients with COVID‐19 is long, and includes antipyretic non‐steroidal anti‐inflammatory drugs (NSAIDs) (eg acetaminophen), traditional Chinese herbal medications (eg bitter apricot seeds), antibiotics (eg azithromycin), immune modulators (eg tocilizumab, hydroxychloroquine) and anti‐viral medications (eg lopinavir/ritonavir, remdesivir). Indeed, the reported histopathological changes—in particular, microvascular steatosis and mild hepatic inflammation in these cases—are also consistent with drug‐induced liver injury.^2^, ^6^, ^24^ A study from Shenzhen, China, analysed the association of abnormal liver function tests with the use of drugs in 417 hospitalized patients with COVID‐19. While antibiotics, Chinese herbal medications and NSAIDs showed a non‐significant tendency towards an increased risk of abnormal liver function tests (odds ratio 2.15 [*P* > .05], 2.21 [*P* > .05] and 1.69 [*P* > .05], respectively), the risk was significantly increased by the use of lopinavir/ritonavir (odds ratio 4.44, *P* < .01).^6^ Immune modulators and remdesivir were not evaluated. Although lopinavir and ritonavir have been discontinued in many centres owing to alack of efficacy,^30^ drugs probably play a role in liver injury in COVID‐19.

## 4 COVID‐19 IN PATIENTS WITH PRE‐EXISTING LIVER DISEASE

A multicentre study with 2780 patients hospitalized for SARS‐CoV‐2 infection in 34 healthcare centres across the USA analysed the influence of pre‐existing liver disease on liver function tests and mortality in COVID‐19. A total of 250 (9%) of the patients had pre‐existing liver disease, usually fatty liver disease or non‐alcoholic steatohepatitis (42%), chronic viral hepatitis (21%), alcoholic liver disease (8%), primary sclerosing cholangitis and primary biliary cholangitis (8%) or autoimmune hepatitis (4%). Twenty‐four per cent were found to have cirrhosis. The mean aminotransferases levels were elevated from baseline after the diagnosis of COVID‐19 in patients with and without pre‐existing liver disease, with a tendency towards increased peak aminotransferase levels in patients with pre‐existing liver disease. The risk of mortality from COVID‐19 was significantly increased in patients with pre‐existing liver disease compared to those without (risk ratio 3, *P* = .001), especially in those with cirrhosis (risk ratio 4.6, *P* < .001).^31^ A large study from Great Britain with a cohort of 20 133 hospitalized patients showed that pre‐existing liver disease is not a predisposing factor for the development of COVID‐19 because only 1.6% of the patients had mild and 1.8% moderate or severe pre‐existing liver conditions, while chronic cardiac disease, diabetes and obesity were found in 30.9%, 28.1% and 10.5% respectively.^32^ However, the increased risk of a fatal outcome of COVID‐19 in patients with pre‐existing liver disease was confirmed in a large survey including more than 17 million cases. Chronic liver disease resulted in a hazard ratio of 1.75 for COVID‐19‐related death (95% confidence interval 1.15‐2.03).^33^ Liver function tests were not assessed in either study. A closer look at specific patient groups is interesting:

### 4.1 Obesity and non‐alcoholic fatty liver disease (NAFLD)

Individuals with a poorer prognosis of COVID‐19 are typically older (>60) with metabolic co‐morbidities such as obesity (body mass index >30 kg/m^2^) and diabetes, a profile which is similar to those at increased risk of NAFLD.^33^, ^34^ A study from 2 COVID‐19 hospitals in China compared liver function tests and clinical outcome in patients with (n = 47) and without (n = 155) NAFLD. Patients with NAFLD had a higher risk of progression to severe COVID‐19 (45% vs 7%, *P* < .0001), a longer viral shedding time (17.5 ± 5.2 days vs 12.1 ± 4.4 days, *P* < .0001) and a higher likelihood of abnormal liver function tests from admission to discharge (70% vs 11.1%, *P* < .0001) compared to those without NAFLD.^35^ Almost all liver injury was mild with a hepatocellular pattern. Another study from China reported a >2‐fold higher prevalence of severe COVID‐19 in patients with NAFLD compared to those without NAFLD but only when they were under 60 years old and even after adjustment for possibly confounding factors such as being overweight, diabetes and hypertension (odds ratio 2.67, *P* = .03). In contrast, NAFLD was not associated with the severity of COVID‐19 in elderly patients (> 60 years old).^36^ The authors suggest that hepatic and systemic immune responses caused by NAFLD could increase the severity of the cytokine storm in younger patients with COVID‐19. In the elderly, other comorbidities such as chronic cardiac disease are more prevalent and any association with NAFLD might be masked by influence of the former.^36^ Obesity is characterized by low‐grade chronic inflammation with increased serum levels of pro‐inflammatory cyokines such as IL‐6 (which can favour macrophage activation and development of the cytokine storm in COVID‐19), and a specific immune dysfunction with impaired secretion of antiviral type I interferons (which probably increases the susceptibility to respiratory viral infections such as COVID‐19).^37^ The adipose tissue of obese patients is thought to express high levels of ACE2 and, thus potentially functions as SARS‐CoV‐2 reservoir with prolonged viral shedding time.^37^ SARS‐CoV‐2 infection and the related hyperinflammatory syndrome could act as “second hit” to a simple fatty liver and trigger “acute‐on‐chronic” steatohepatitis (NASH) with elevated aminotransferases.^34^ Moreover, hepatic expression of ACE2 was strongly upregulated in a high‐fat diet‐induced NASH model in rodents,^16^ possibly increasing hepatic susceptibility to SARS‐CoV‐2 infection in patients with NAFLD or NASH. However, the association of NAFLD and hepatic expression of SARS‐CoV‐2 critical entry proteins, such as ACE2 and TMPRSS2, a host cell serine protease which cleaves the SARS‐CoV‐2 spike protein and mediates fusion of host cellular and viral membranes, are controversial. No upregulation was found in a microarray data set comparing 12 lean and 16 obese patients without NAFLD with 9 patients with simple steatosis and 17 patients with biopsy‐proven NASH.^38^ In contrast, hepatic mRNA expression of ACE2 and TMPRSS2 was low in obese subjects without liver injury (n = 17) or with simple steatosis (n = 57) but significantly increased in obese patients with NASH (*P* < .01 and *P* < .05) and correlated with the NAFLD activity score (NAS) (*P* = .017 and *P* = .003, respectively).^39^ Finally, obesity is related to hypercoagulation, mainly as a result of higher plasma concentrations of prothrombotic factors such as factor VII, fibrinogen and von Willebrand factor. This probably fosters microvascular thrombosis formation with ischemia‐induced hypoxic liver damage.^37^ In conclusion, an inherent immune activation and a tendency towards hypercoagulation are potential causes of the poorer prognosis of COVID‐19 and a higher risk of abnormal liver function tests during the course of COVID‐19 in patients with obesity and NAFLD.

### 4.2 Cirrhosis and liver transplantation

**FIGURE 3:**
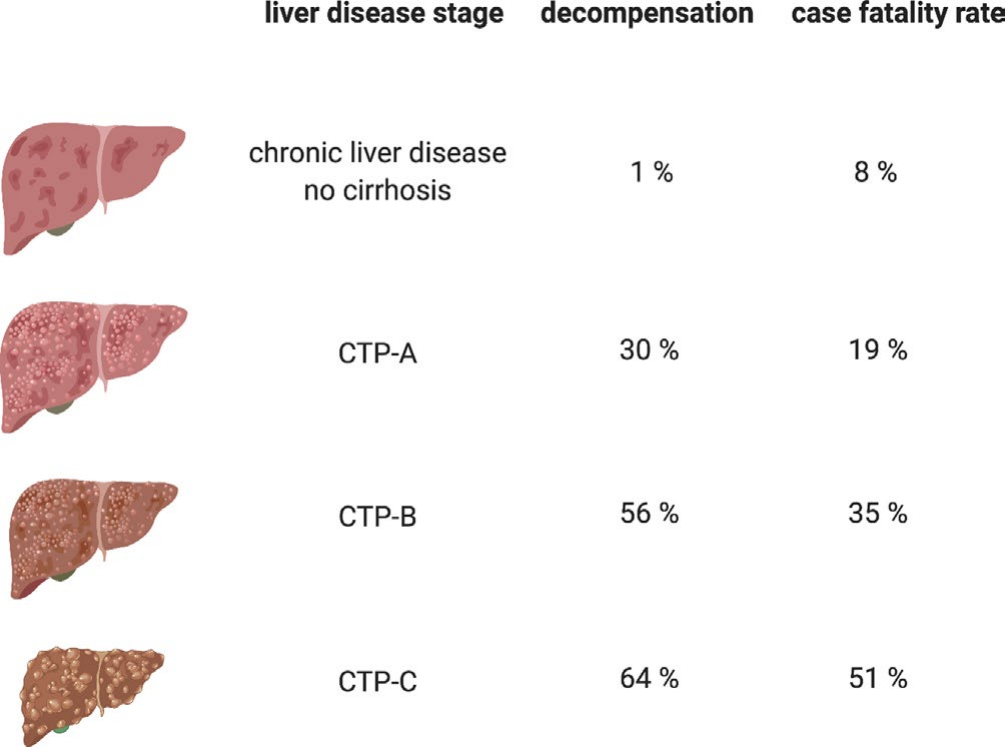
Acute hepatic decompensation and fatality rates in hospitalized patients with chronic liver disease and COVID‐19. Decompensation included new or worsening ascites (28%), hepatic encephalopathy (27%), spontaneous bacterial peritonitis (3%) and variceal haemorrhage (3%). CTP, Child‐Turcotte‐Pugh. Adapted from Ref. [42] [Correction added on 5 August 2021, after first online publication: Figure 3 image has been replaced.]

Pre‐existing cirrhosis with cirrhosis‐associated immune dysfunction and immunosuppressive therapy after liver transplantation could favour liver‐related complications, more severe courses of COVID‐19 and higher mortality rates. A retrospective analysis of 50 patients with cirrhosis from 9 hospitals in Lombardy, Italy, showed that liver function declined in patients with cirrhosis and COVID‐19 upon hospital admission compared to the last visit before SARS‐CoV‐2 infection. Serum ALAT (31 vs 54 IU/L, *P* = .024), ASAT (33 vs 48 IU/L, *P* = .176), bilirubin (1.3 vs 1.8 mg, *P* = .026) and international normalized ratio (INR) (1.2 vs 1.3, *P* = .042) increased and serum albumin levels (3.4 vs 2.8 g/dL, *P* =.0003) decreased, thus influencing both the Child‐Turcotte‐Pugh (CTP) and Model for End‐Stage Liver Disease (MELD) scores. The distribution of CTP scores shifted towards class C (*P* = .05) and the proportion of patients with MELD ≥ 15 increased from 13% to 26% (*P* = .037). Acute liver injury (ALAT > 30 IU/L for men or >19 IU/L for women) developed in 45% of the patients with previously persistent normal ALAT levels, while 12% experienced a hepatic flare (ALAT ≥ 5× ULN). The 30‐day cumulative probability of mortality was significantly higher in the COVID‐19 cohort than in 47 patients with cirrhosis hospitalized for acute liver decompensation as a result of bacterial infection (34% vs 17%, *P* = .03).^40^ A large‐scale international open online reporting study coordinated by the COVID‐Hep registry compared COVID‐19 mortality and liver injury in chronic liver disease patients with (n = 386) and without (n = 359) cirrhosis. Overall mortality was 32% in patients with cirrhosis and 8% in those without (*P* < .001). The stage of liver disease was the most important determinant of outcome because mortality in patients with cirrhosis increased according to CTP class (A 19%, B 35% and C 51%) (Figure 3). Fifty‐five per cent of patients with cirrhosis developed one or more acute‐on‐chronic liver failure (ACLF) criteria, defined by the Clif consortium.^41^ Mortality in patients with ACLF was higher than in those without (46% vs 14%, *P* < .001). Respiratory failure was the most common cause of death (71%), even in patients with ALCF. Alcohol‐related liver disease was the only aetiology that was an independent risk factor of death from COVID‐19 (odds ratio 3.11, *P* < .001).^42^ Another multicentre cohort study from the COVID‐Hep registry compared the clinical outcomes of SARS‐CoV‐2 infection in patients who had undergone liver transplantation (LT) for end‐stage liver disease (LT cohort, n = 151) with a matched comparison cohort (non‐LT cohort, n = 627). Ninety‐nine per cent of the patients in the LT cohort were taking immunosuppressive drugs when SARS‐CoV‐2 infection was diagnosed: tacrolimus (84%), prednisolone (44%), mycophenolate (51%), azathioprine (9%), cyclosporin (5%) and sirolimus (5%). The median time from liver transplantation was 5 years. The groups did not differ for hospitalization (82% vs 76%, *P* = .106) or the need for intensive care (31% vs 30%, *P* = .837). The percentage of patients who died in the LT cohort was lower than that in the non‐LT cohort (19% vs 27%, *P* = .046). The main cause of death in both groups was respiratory failure (75% and 89%). The biological age (odds ratio 1.06 per 1 year increase, *P* = .031) but not the time since LT or immunosuppressive medication was associated with mortality in the LT cohort. The LT and non‐LT cohorts did not differ in the frequency of mild liver injury (ALAT > 40 IU/L, 30% vs 28%, *P* = .734), moderate liver injury (ALAT > 80 IU/L, 16% vs 14%, *P* = .662) or severe liver injury (ALAT > 200 IU/L, 8% vs 4%, *P* = .052).^43^ However, it is important to note that the median time from LT was 5 years, thus these results cannot be applied to patients who acquire SARS‐CoV‐2 infection in the perioperative period.^44^ In a study from Spain, the inflammatory response after SARS‐CoV‐2 infection in solid organ transplant recipients (kidney, lung, liver, n = 46) and a matched control group (n = 166) was analysed. The inflammatory response in solid organ transplant recipients with COVID‐19 was not stronger (according to lymphocyte count, IL‐6 and CRP) than in the control group. In contrast, median IL‐6 after 7 days of admission (231.4 vs 534.6 pg/ml, *P* = .433) and the incidence of acute respiratory distress syndrome (ARDS) (19.6% vs 27.1%, *P* = .06) were lower in transplant recipients than in the control group.^45^ The authors suggest that immunosuppressive medication in solid organ transplant recipients might limit the inflammatory response and protect these patients from hyperinflammation and ARDS development in COVID‐19. Unlike the COVID‐Hep registry study,^43^ this study also included patients with a shorter time after transplantation (<3 months 6.7%, 3‐6 months 6.7% and >12 months 87%).^45^ In conclusion, although a reporting bias may have affected the COVID‐Hep registry data, the overall evidence clearly suggests that cirrhosis strongly increases the risk of COVID‐19‐related liver injury and mortality with a positive correlation with the stage of cirrhosis. When hepatic function is restored by LT, the risk of liver injury and mortality return to that of the general population emphasizing the close association between chronic liver disease and an adverse outcome of COVID‐19.^42^ Clear data on the outcome of COVID‐19 and post‐operative transplant engraftment (<6 months) are lacking.

### 4.3 Autoimmune liver disease and chronic viral hepatitis

Studies on the influence of autoimmune liver diseases and chronic viral hepatitis without cirrhosis on COVID‐19‐related liver injury and outcome are limited. Reports from Northern Italy, China and Belgium suggest that the case‐to‐infection ratio is not higher in patients with autoimmune hepatitis (AIH), primary biliary cholangitis (PBC) or primary sclerosing cholangitis (PSC) without cirrhosis with a fairly favourable outcome for SARS‐CoV‐2 infection in these patients.^1^, ^46^, ^47^ In a case series in 10 hospitalized patients with AIH and symptomatic SARS‐CoV‐2 infection, liver function tests remained normal throughout the hospital stay with a stable immunosuppression regimen, and improved in 2 cases with the onset of acute AIH and high‐dose steroid induction therapy.^48^ The authors concluded that reduction in immunosuppression during COVID‐19 could be harmful, as (i) patients with AIH are at risk of relapse when immunosuppression is reduced, and (ii) immunosuppressive medication could counterbalance COVID‐19‐driven hyperinflammation. Little is known about the impact of viral hepatitis without associated cirrhosis on outcome and liver function tests in COVID‐19. A multicentre study from China suggests that acute or chronic hepatitis B virus (HBV) infection does not affect the outcome of COVID‐19, as 22 (95%) of 23 included patients with acute or chronic HBV infection (defined as positive hepatitis B surface antigen) showed a non‐severe course of COVID‐19.^5^ A survey from Spain showed that the use of immunosuppressive drugs (eg IL‐6 receptor antagonists or corticosteroids) for the treatment of patients with severe hyperinflammatory syndrome in COVID‐19 and resolved chronic HBV infection does not increase the risk of HBV reactivation.^49^ However, liver function tests and the outcome of COVID‐19 were not assessed. Of note, the significant strain of COVID‐19 on national healthcare systems around the world in 2020 has disrupted progress in the global hepatitis C virus (HCV) elimination program, which could result in more than 44 800 cases of hepatocellular carcinoma and 72 300 HCV‐related deaths.^50^ This analysis shows that COVID‐19 extends the direct morbidity and mortality associated with exposure and infection.^50^ In conclusion, autoimmune liver diseases without cirrhosis do not seem to increase the risk of COVID‐19‐related liver injury and mortality, even though the number of patients evaluated is small. The influence of chronic viral hepatitis on liver function tests and the severity of COVID‐19 remains to be clarified.

## 5 CONCLUDING REMARKS

Although evidence is limited to hospitalized patients, abnormal liver function tests in COVID‐19 are common, especially in patients with severe disease. This probably reflects multifactorial mechanisms of liver injury. Initial abnormalities include elevated aminotransferases, probably mainly because of hypoxic hepatocellular damage. The tropism of SARS‐CoV‐2 is broad and includes hepatocytes and cholangiocytes. Nevertheless, cases of acute hepatitis are rare. A delayed cholestatic liver biochemistry pattern can develop in patients with critical COVID‐19, and its close association with inflammatory response markers supports underlying cytokine‐induced molecular mechanisms. Well‐controlled pre‐existing chronic liver disease without cirrhosis is not associated with a risk of abnormal liver function tests or a fatal outcome, except in patients with pre‐existing NAFLD, who have a higher risk of progression to severe COVID‐19 and likelihood of abnormal liver function tests. Cirrhosis strongly increases the risk of COVID‐19‐related liver injury and mortality, with a clear positive correlation with the stage of cirrhosis. The SARS‐CoV‐2 infection‐related risk of liver injury and mortality is similar to that of the general population following liver transplantation, although more data on its effect in the early postoperative period are needed. Optimal treatment and compensation of chronic liver diseases are highly important in this period of limited healthcare resources to prevent severe courses of COVID‐19 in these patients.^51^, ^52^

## CONFLICT OF INTEREST

The authors declare no conflict of interest with regard to this work.
